# Possible discrepancy of HbA1c values and its assessment among patients with chronic renal failure, hemodialysis and other diseases

**DOI:** 10.1007/s10157-015-1110-6

**Published:** 2015-04-01

**Authors:** Kaori Inoue, Atsushi Goto, Miyako Kishimoto, Tetsuro Tsujimoto, Ritsuko Yamamoto-Honda, Hiroshi Noto, Hiroshi Kajio, Yasuo Terauchi, Mitsuhiko Noda

**Affiliations:** Department of Endocrinology, Diabetes, and Metabolism, Center Hospital, National Center for Global Health and Medicine, Tokyo, 162-8655 Japan; Department of Diabetes Research, National Center for Global Health and Medicine, 1-21-1 Toyama, Shinjuku-ku, Tokyo, 162-8655 Japan; Department of Endocrinology and Metabolism, Yokohama City University Graduate School of Medicine, Yokohama, 236-0004 Japan

**Keywords:** Glycated hemoglobin, Glycated albumin, Chronic renal failure

## Abstract

**Background:**

Glycated hemoglobin (HbA1c) and glycated albumin (GA) are frequently used as glycemic control markers. However, these markers are influenced by alterations in hemoglobin and albumin metabolism. Thus, conditions such as anemia, chronic renal failure, hypersplenism, chronic liver diseases, hyperthyroidism, hypoalbuminemia, and pregnancy need to be considered when interpreting HbA1c or GA values. Using data from patients with normal albumin and hemoglobin metabolism, we previously established a linear regression equation describing the GA value versus the HbA1c value to calculate an extrapolated HbA1c (eHbA1c) value for the accurate evaluation of glycemic control. In this study, we investigated the difference between the measured HbA1c and the eHbA1c values for patients with various conditions.

**Methods:**

Data sets for a total of 2461 occasions were obtained from 731 patients whose HbA1c and GA values were simultaneously measured. We excluded patients with missing data or changeable HbA1c levels, and patients who had received transfusions or steroids within the previous 3 months. Finally, we included 44 patients with chronic renal failure (CRF), 10 patients who were undergoing hemodialysis (HD), 7 patients with hematological malignancies and a hemoglobin level of less than 10 g/dL (HM), and 12 patients with chronic liver diseases (CLD).

**Results:**

In all the groups, the eHbA1c values were significantly higher than the measured HbA1c values. The median difference was 0.75 % (95 % CI 0.40–1.10 %, *P* for the difference is <0.001) in the CRF group, 0.80 % (95 % CI 0.30–1.65 %, *P* for the difference is 0.041) in the HD group, 0.90 % (95 % CI 0.90–1.30 %, *P* for the difference is 0.028) in the HM group, and 0.85 % (95 % CI 0.40–1.50 %, *P* for the difference is 0.009) in the CLD group.

**Conclusions:**

We found that the measured HbA1c values were lower than the eHbA1c values in each of the groups.

## Introduction

Glycated hemoglobin (HbA1c) and glycated albumin (GA) are frequently used as glycemic control markers. HbA1c is used as the gold standard index of glycemic control in clinical practice for diabetes treatment [1]. Since the lifespan of erythrocytes is approximately 120 days, HbA1c reflects the plasma glucose levels over the past few months. The metabolic turnover of albumin is faster than hemoglobin, with a lifespan of approximately 17–23 days. Accordingly, GA is used as an index of short-term glycemic control [2].

Although these glycemic control markers are well correlated with blood glucose levels, HbA1c is influenced by alterations in hemoglobin metabolism and GA is influenced by alterations in albumin metabolism. In clinical practice, conditions such as anemia, chronic renal failure, hypersplenism, chronic liver diseases, hyperthyroidism, hypoalbuminemia, and pregnancy need to be considered when interpreting HbA1c or GA values.

In a previous study, we developed a linear regression equation describing the GA value versus the HbA1c value among participants without altered albumin metabolism or hemoglobin metabolism, to calculate an extrapolated HbA1c (eHbA1c) value for the accurate evaluation of glycemic control [3].

We often encounter patients with conditions affecting the turnover of either HbA1c or GA. In such patients, the measured HbA1c and GA values are likely to diverge from the equation. Earlier studies have evaluated the associations between mean blood glucose levels, HbA1c values, and GA values in patients on dialysis or patients with chronic liver diseases or hemolytic anemia [4–6]. However, the impact of each condition affecting the turnover of either HbA1c or GA on the direction and magnitude of the discrepancy between the measured HbA1c and eHbA1c, which is the equation developed in patients who were free of such conditions is not well understood. In this study, we investigated the differences between the measured HbA1c and the eHbA1c values in patients with various conditions.

## Materials and methods

**Fig. 1:**
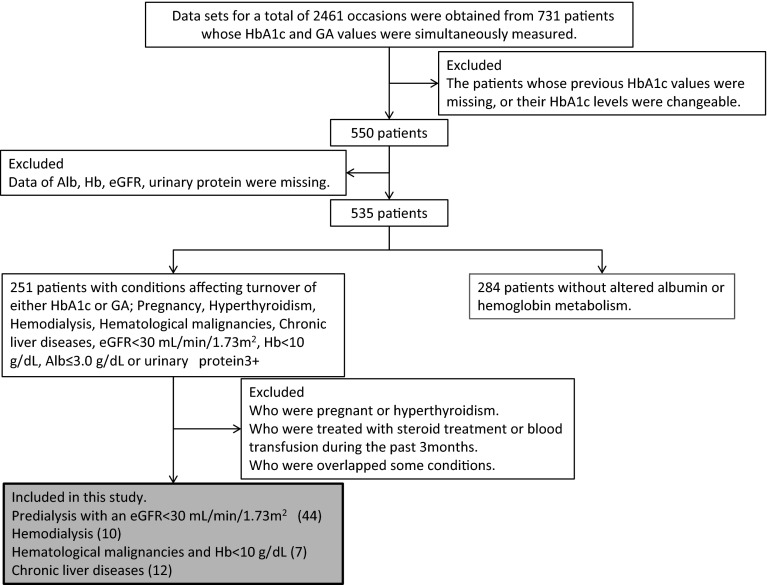
Flow diagram depicting the study. Data sets for a total of 2461 occasions were obtained from 731 patients (including non-diabetes patients) whose HbA1c and GA values were simultaneously measured. If these values were measured in the patients on more than one occasion, the data set containing the smallest HbA1c value was selected. We then excluded patients whose previous HbA1c values were missing or whose HbA1c levels were changeable, selecting 550 patients. We excluded patients without albumin, hemoglobin or eGFR data, and patients who had been treated with transfusions or steroids within the previous 3 months. Finally, we included 44 predialysis patients with an eGFR of less than 30 mL/min/1.73 m^2^ (CRF), 10 patients who were undergoing hemodialysis (HD), 7 patients with hematological malignancies and their hemoglobin level of less than 10 g/dL (HM), and 12 patients with chronic liver diseases (CLD). We further excluded patients who had combinations of these diseases, since the aim of this study was to investigate the impact of each condition affecting the turnover of either HbA1c or GA on the direction and magnitude of the discrepancy

A flow diagram depicting this study is shown in Fig. 1. We retrospectively analyzed the medical charts of patients attending the National Center for Global Health and Medicine (Tokyo, Japan) during 2011, and selected data sets for a total of 2461 occasions from 731 patients (including non-diabetes patients) whose HbA1c and GA values were simultaneously measured. If these values were measured in a single patient on more than one occasion, we selected the data set containing the smallest HbA1c value.

We excluded patients whose previous HbA1c values were missing or whose HbA1c levels were changeable and selected 550 patients. We then excluded patients without albumin, hemoglobin or eGFR data, and patients who had been treated with transfusions or steroids within the previous 3 months. Finally, we included 44 predialysis patients with an eGFR of less than 30 mL/min/1.73 m^2^ chronic renal failure (CRF), 10 patients who were undergoing hemodialysis (HD), 7 patients with hematological malignancies and their hemoglobin level of less than 10 g/dL (HM), and 12 patients with chronic liver diseases (CLD). We further excluded patients who had combinations of these diseases, since the aim of this study was to investigate the impact of each condition on the turnover of either HbA1c or GA, as well as the direction and magnitude of the discrepancy. We did not include patients who were pregnant or who had hyperthyroidism because the data was insufficient for an analysis.

HbA1c was measured using high-performance liquid chromatography (HPLC) (ARKRAY ADAMS-A1C HA-8160; Kyoto, Japan) and was corrected to the National Glycohemoglobin Standardization Program (NGSP) values [7]. GA was measured using an enzymatic method with albumin-specific proteinase, ketoamine oxidase, and an albumin assay reagent (Lucica GA-L; Asahi Kasei Pharma Co., Tokyo, Japan) using an autoanalyzer (Hitachi 770; Hitachi Instruments Service Co., Tokyo, Japan). Each patient was assessed for clinical features such as age, sex, height, body weight, body mass index, blood and urine sample data, history and duration of diabetes mellitus, medications, and complications based on the data contained in the medical records.

This study was approved by the institutional ethical committee of the National Center for Global Health and Medicine (approval number: 1141) and was performed in accordance with the Declaration of Helsinki.

### Statistical analysis

We performed the statistical analyses using Stata/IC 11. Data for the patient characteristics are shown as the mean ± SD. To investigate the difference between the eHbA1c and measured HbA1c values, we calculated 95 % confidence intervals (CI) of the median of the difference using a bootstrap method (2000 bootstraps), and determined the *P* values for the difference using the Wilcoxon signed-rank test.

## Results

The clinical characteristics in each group are shown in Table 1. Patients in the HM group were less likely to have diabetes than patients in the other groups. The HbA1c, GA, hemoglobin, albumin, and eHbA1c levels were lower in the HM group than the other groups. The eGFR levels in the CRF and HD groups were lower than the other groups. Patients in the CRF and HD groups tended to have proteinuria and require erythropoietin or iron preparations.

**Table 1:** Clinical characteristics in each groups

	Predialysis with an eGFR <30 mL/min/1.73 m^2^ (*n* = 44)	Hemodialysis (*n* = 10)	Hematological malignancies and Hb <10 g/dL (*n* = 7)	Chronic liver diseases (*n* = 12)
Men (*n*)	35	8	5	6
Age (years)	66.8 ± 12.0	67.8 ± 11.7	69.3 ± 18.2	71.5 ± 10.3
HbA1c (%)	6.8 ± 1.3	6.4 ± 0.9	5.7 ± 0.5	7.1 ± 0.8
GA (%)	20.8 ± 5.7	19.7 ± 4.5	16.1 ± 2.0	22.9 ± 4.4
Hb (g/dL)	11.3 ± 1.8	10.9 ± 1.6	8.7 ± 0.8	12.1 ± 1.6
Alb (g/dL)	3.7 ± 0.5	3.6 ± 0.9	2.9 ± 0.7	3.7 ± 0.4
eHbA1c (%)	7.5 ± 1.2	7.3 ± 1.0	6.4 ± 0.4	7.9 ± 1.0
eGFR (mL/min/1.73 m^2^)	16.6 ± 7.8	–	123.3 ± 104.1	69.0 ± 14.4
Diabetes (*n*)	43	9	2	11
Urinary protein3+ (*n*)	13	4	0	0
Using erythropoietin (*n*)	19	6	0	0
Using iron preparation (*n*)	7	2	0	1
				Mean ± SD

**Fig. 2:**
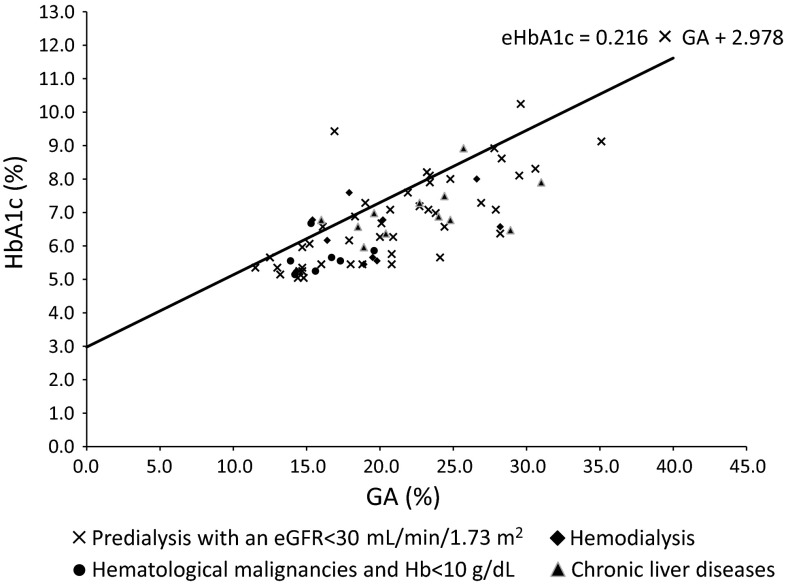
Scatter plots for HbA1c values versus GA values in each group. In our previous study, we established the following equation: ${\text{eHbA1c = 0}} . 2 1 6 \times {\text{GA + 2}} . 9 7 8$. Scatter plots for the HbA1c values versus the GA values are shown for each group with a line for the equation. In all the groups, the eHbA1c values tended to be higher values than the measured HbA1c levels

In our previous study, we established the following equation: ${\text{eHbA1c = 0}} . 2 1 6 \times {\text{GA + 2}} . 9 7 8$ [3]. Figure 2 shows scatter plots for the HbA1c values versus the GA values for each group with a line for the equation.

In all the groups, the eHbA1c values (i.e., the line for the equation in Fig. 2) tended to be higher than the measured HbA1c levels. We further analyzed the medians of the differences between the eHbA1c and measured HbA1c values, and calculated the corresponding 95 % CI and *P* values for each group (Table 2). In all the groups, the eHbA1c values were significantly higher than the measured HbA1c levels. The median of the difference was 0.75 % (95 % CI 0.40–1.10 %, *P* for the difference is <0.001) in the CRF group, 0.80 % (95 % CI 0.30–1.65 %, *P* for the difference is 0.041) in the HD group, 0.90 % (95 % CI 0.90–1.30 %, *P* for the difference is 0.028) in the HM group, 0.85 % (95 % CI 0.40–1.50 %, *P* for the difference is 0.009) in the CLD group.

**Table 2:** The medians of the difference between eHbA1c and measured HbA1c values in each groups

	The median of the difference between eHbA1c and measured HbA1c values (%)	95 % CI	*P* values
Predialysis with an eGFR <30 mL/min/1.73 m^2^ (*n* = 44)	0.75	0.40–1.10	<0.001
Hemodialysis (*n* = 10)	0.80	0.30–1.65	0.041
Hematological malignancies and Hb <10 g/dL (*n* = 7)	0.90	0.90–1.30	0.028
Chronic liver diseases (*n* = 12)	0.85	0.40–1.50	0.009

## Discussion

In this study, we calculated the eHbA1c value using an equation for each of the several groups of patients suffering from various diseases, and investigated the difference from the measured HbA1c values. Few studies have investigated the difference between estimated values and actual measurements of HbA1c.

The patients were classified into 4 groups as follows: 44 patients with chronic renal failure, 10 patients undergoing hemodialysis, 7 patients suffering from hematological malignancies and who had a hemoglobin level of less than 10 g/dL, and 12 patients who were suffering from chronic liver diseases. In all of the groups, the eHbA1c values were significantly higher than the measured HbA1c values. These results suggested that the measured HbA1c values in these groups may be underestimated in clinical practice.

In cases with chronic renal failure, renal anemia lowers the HbA1c values because the lifespan of the erythrocytes is shortened. The HbA1c and eGFR values are reportedly correlated with the lifespan of the erythrocytes in patients with diabetic nephropathy [8]. It has also been reported that the values of HbA1c are underestimated in patients with diabetic nephropathy undergoing peritoneal dialysis or hemodialysis [9]. Furthermore, the HbA1c values in patients who were treated with erythropoietin were lower than those patients who were not treated, since the life span of the erythrocytes is shortened [10]. Because renal anemia is unlikely to affect the GA value, GA may be useful in patients with renal anemia. Although HbA1c has been commonly measured, several professional societies (e.g., the Japanese Society for Dialysis Therapy [11] ) now recommend GA measurements for such patients. Our findings further suggest that eHbA1c may be a useful marker for the evaluation of glycemic control in patients with CRF or HD. However, a careful consideration is required in patients with diabetic nephropathy with marked proteinuria. The GA values are affected by the increased turnover of albumin metabolism and tend to decrease independent of glycemic state in patients with marked proteinuria [12], indicating their possible limited ability to evaluate glycemic control in such patients. Because the number of patients with marked proteinuria was relatively small in the present study, further studies are needed to clarify whether eHbA1c or GA is more useful than HbA1c in such patients.

In this study, we investigated 7 patients who were suffering from hematological malignancies and who had a hemoglobin level of less than 10 g/dL. Both the measured HbA1c and the eHbA1c levels in the HM group were lower than those in the other groups. The lower frequency of patients with diabetes in the HM group may explain the lower GA and eHbA1c levels. HbA1c values are known to be low, relative to the glucose levels in patients with hemolytic anemia because the lifespan of the erythrocytes is shortened in patients with this condition [6]. Moreover, in patients with iron deficiency anemia, the HbA1c values tend to be higher than in healthy individuals but decrease after iron treatment [13]. Although the mechanisms remain to be investigated, the altered lifespan of erythrocytes may partially explain the difference between the measured HbA1c and eHbA1c levels in the HM group observed in this study.

In chronic liver diseases, such as chronic hepatitis and liver cirrhosis, hypersplenism lowers the HbA1c values because of the shortened lifespan of the erythrocytes, whereas, it raises the GA values because of reduced albumin synthesis and the prolonged half-life of serum albumin [5, 14]. Although neither marker reflects the plasma glucose control status accurately, we found the eHbA1c values were significantly higher than the measured HbA1c values in the CLD group.

Our study had several limitations. First, we retrospectively selected patients in whom simultaneous HbA1c and GA measurements had been obtained. Thus, a selection bias may exist. We excluded the patients, whose previous HbA1c values were missing or their HbA1c levels were changeable, but we couldn’t exclude the patients who had become good control over past few weeks. Second, as the data were collected from a single hospital and the GA values were not standardized, the present results might not be directly applicable to other hospitals. Third, the small sample size might limit the applicability of the findings. In clinical situation, patients with various conditions affect the GA values, so we should take consideration to use the equation of the eHbA1c.

In conclusion, we found that the measured HbA1c values were lower than the eHbA1c values in groups of patients with chronic renal failure, who were undergoing hemodialysis, suffering from hematological malignancies and had a hemoglobin level of less than 10 g/dL, and who had chronic liver diseases.
